# Luteolin Protects Against Acrylamide‐Induced Cellular Toxicity in Mouse Leydig Cells

**DOI:** 10.1002/fsn3.70621

**Published:** 2025-07-14

**Authors:** Ertan Calmaz, Banu Orta‐Yilmaz, Yasemin Aydin

**Affiliations:** ^1^ Department of Biology, Institute of Graduate Studies in Science and Engineering Istanbul University Istanbul Turkey; ^2^ Department of Biology, Faculty of Science Istanbul University Istanbul Turkey

**Keywords:** acrylamide, apoptosis, Leydig cells, luteolin, oxidative stress

## Abstract

Luteolin (Lut), a natural flavonoid, possesses the ability to protect the organism from reactive oxygen species (ROS) by modulating intracellular and extracellular signals. This study was conducted to investigate whether Lut exhibits a protective effect against acrylamide (Acr)‐induced reproductive toxicity and to reveal the mechanisms underlying Leydig cell damage, a model cell in the male reproductive system. TM3 mouse Leydig cells were treated with Acr (1 mM) and/or Lut (1 and 5 μM) for 24 h. To determine the potential protective effects of Lut against the toxicity caused by Acr on Leydig cells, cell viability, combination index, and lactate dehydrogenase activity were examined for cytotoxicity. The effect of Lut on oxidative stress parameters in Acr‐induced cellular damage was revealed by evaluating lipid peroxidation, total ROS, glutathione, and antioxidant enzymes (SOD, CAT, and GPx). In addition, we evaluated the expression levels of genes related to apoptosis (*Trp53*, *Casp3*, *Bax*, and *Bcl2*) and the apoptotic rate in the Acr and/or Lut groups. The findings indicated that Lut inhibited the cytotoxicity elevated by Acr in Leydig cells. The evaluation of the combination index in the groups treated with Lut and Acr together revealed that Lut exhibited an antagonistic effect. Furthermore, it was revealed that while Acr induced oxidative damage and apoptosis, Lut reversed these effects and protected Leydig cells from cellular damage. Also, Lut inhibited the mRNA expressions of *Trp53*, *Casp3*, and *Bax*, which were upregulated by Acr. Consequently, Lut may serve as a potential antioxidant molecule in promoting cell survival by mitigating oxidative damage and apoptosis induced by Acr.

## Graphical abstract

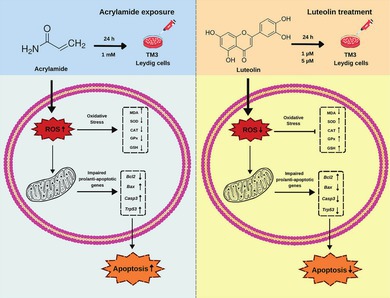

## 1 Introduction

Flavonoids are essential in mitigating the detrimental effects of hazardous substances on the body due to their antioxidant properties. Flavonoids, prevalent in fruits and vegetables, are a category of plant secondary metabolites that confer significant health benefits due to their protective properties (Panche et al. 2016). Luteolin (Lut), a member of the flavone subclass of flavonoids, often referred to as 3′,4′,5′,7′‐tetrahydroxyflavone, is a naturally occurring chemical found in various foods (Lin et al. 2008). Lut, found in plants in its glycosylated form, 7‐O‐ß‐glucoside, is biologically activated in the body by the intestinal mucosa and absorbed into the systemic circulation (Hayasaka et al. 2018). The maximum concentration reached in plasma by dietary Lut after oral intake of 14.3 mg/kg was calculated to be approximately 1.97 μg/mL (Luo et al. 2017). This amount shows that 14% of Lut is effectively found in plasma. Lut provides protection against chemical and natural toxic substances with its antioxidant, anti‐inflammatory, and antiapoptotic properties (Mahdiani et al. 2022). Lut acts as a ROS scavenger by facilitating unpaired electrons and inhibiting ROS‐generating oxidases, including xanthine oxidase, which contribute to the production of superoxide anion. Furthermore, it might mitigate oxidative stress by enhancing enzymatic antioxidants including glutathione‐S‐transferase (GST), superoxide dismutase (SOD), and catalase (CAT), as well as by chelating transition metals that induce ROS generation (Lin et al. 2008). A study showed that Lut alleviated oxidative stress by suppressing the p38 MAPK/NF‐kß signaling pathway in endothelial cells exposed to hydrogen peroxide. Lut protected cells against apoptosis by regulating the expression levels of apoptotic proteins and suppressed mitochondrial dysfunction (Chen et al. 2020). In Wistar rats exposed to lead acetate, the elevated apoptosis rates decreased with the administration of Lut (AL‐Megrin et al. 2020). However, there is a paucity of research about the ameliorative effects of Lut, which has been the focus of attention in recent years for its potent antioxidant properties in acrylamide (Acr)‐induced toxicity.

Acr, which is not naturally found in foods, can negatively affect human health by causing toxicity. Acr is a food contaminant that is released as a result of cooking processes such as frying, grilling, roasting, and baking and is found in various concentrations in the foods we consume daily (Starowicz and Zieliński 2019). Acr is particularly generated during the Maillard reaction between reducing sugars and the amino acid asparagine, especially in carbohydrate‐rich foods such as fried potatoes, bread crust, and coffee (Friedman 2003). The primary route of exposure in humans is dietary intake, although tobacco smoke and occupational exposure also contribute (Tareke et al. 2002). From a food safety perspective, the European Food Safety Authority (EFSA) has concluded that no tolerable daily intake level can be established for Acr, emphasizing that exposure should be kept as low as reasonably achievable (EFSA 2015). Therefore, elucidating the toxicological mechanisms of Acr and developing preventive strategies are of great importance for public health. It is known that Acr has neurotoxic, cytotoxic, genotoxic, and carcinogenic effects on living organisms (Koszucka and Nowak 2019). These effects are largely attributed to its reactive metabolite, glycidamide, which forms covalent adducts with DNA and proteins, leading to genotoxicity and oxidative stress (Dearfield et al. 1988). Furthermore, Acr can increase reactive oxygen species (ROS) production, contributing to cellular oxidative damage and dysfunction (Albalawi et al. 2017). Numerous in vivo and in vitro investigations have examined the impact of Acr on the male reproductive system. Research on male rats has demonstrated that Acr exposure adversely affects seminiferous tubules, diminishes sperm production, disrupts spermatozoa development, elevates the incidence of aberrant sperm formation, and decreases sperm viability (Ma et al. 2011; Wang et al. 2010). In vitro studies with Leydig and Sertoli cells have shown that Acr reduces cell viability and increases cytotoxicity, oxidative stress, and apoptosis (Yilmaz et al. 2017; Zhang et al. 2023). Research indicates that many plant‐derived flavonoids are capable of decreasing the toxicity of Acr in response to Acr‐induced oxidative damage and apoptosis. While numerous studies have demonstrated the impact of flavonoids such as naringenin, quercetin, apigenin, and catechin on Acr toxicity, research on Lut remains quite limited (Baraka et al. 2024; El‐Beltagi and Ahmed 2016; Sengul et al. 2023; Wang et al. 2022). Here, we focused on the protective role of Lut in Acr‐induced cellular damage in mouse Leydig cells. In this context, we evaluated the potential benefits of Lut in reducing Acr‐associated Leydig cell damage by examining apoptosis and oxidative stress parameters.

## 2 Materials and Methods

### 2.1 Chemicals

Acr (purity ≥ 99%, Cas no.: 79‐06‐1) was obtained from Biomatik Corporation (Ontario, Canada). Lut (purity ≥ 98%, Cas no.: 491‐70‐3), Hoechst 33342 (Ho342), hydrogen peroxide (H_2_O_2_), propidium iodide (PI), dimethyl sulfoxide (DMSO), Triton X‐100, Tris, and 2′,7′‐dichlorofluorescin diacetate (DCFH‐DA) were purchased from Sigma‐Aldrich (St. Louis, MO, USA). Dulbecco's modified Eagle's medium/Hams F12 (DMEM/F12) media, fetal bovine serum, horse serum, penicillin–streptomycin solution, phosphate buffer saline (PBS), and trypsin–EDTA were obtained from Wisent Bioproducts (Quebec, Canada).

### 2.2 Cell Culture Condition and Study Design

The TM3 mouse Leydig cell line (Product code: CRL‐1714) was obtained from the American‐Type Culture Collection and transferred to our laboratory. The cells were cultured in DMEM/F12 medium supplemented with 5% horse serum, 2.5% fetal bovine serum, and 1% penicillin–streptomycin solution at 37°C, 5% CO_2_. The Acr (1 mM) concentration used in this study was chosen based on our previous studies (Orta‐Yilmaz 2019; Yilmaz et al. 2017). Although the Acr concentration used in this study (1 mM) may be considered relatively high for in vitro experiments, this dose was deliberately chosen to mimic acute exposure scenarios and to challenge the upper threshold of the protective effects of luteolin (Albalawi et al. 2017; Jiang et al. 2018). In contrast, actual human exposure to acrylamide is considerably lower and occurs chronically through various food sources. However, to evaluate potential mechanisms of toxicity and test the efficacy of protective agents such as luteolin under oxidative stress, high‐dose models are frequently employed in toxicological studies (Song et al. 2013; Sun et al. 2018). Therefore, the selected concentration in this study serves to model an extreme exposure condition, which allows for clearer observation of cytotoxic outcomes and the protective potential of candidate compounds. The luteolin concentrations used in this study (1 and 5 μM) were selected based on the findings of Chen et al. (2020) and our previous publication (Keskin et al. 2025). These concentrations were dissolved in 100% DMSO and diluted in DMEM/F12 medium to yield a final DMSO concentration of 0.05%. Cells were treated with Acr and Lut simultaneously for 24 h. DMSO was used as a solvent at a final concentration of 0.05% in all treatment groups. A vehicle control group containing only 0.05% DMSO was included in the experiments, and no significant differences were observed compared to the untreated control, indicating that DMSO had no observable effect on cell viability or gene expression under the experimental conditions.

### 2.3 Cell Viability and Combination Index (CI)

Cell viability was measured by the conversion of 3‐(4, 5‐dimethylthiazol‐2‐yl)‐2, 5‐diphenyltetrazolium bromide (MTT) into purple formazan crystals by the enzymatic activity of mitochondrial dehydrogenase in metabolically active cells. Briefly, TM3 Leydig cells were seeded into a 96‐well plate with 5 × 10^3^ cells per well, and then the MTT kit protocol was applied (Cat. No: 11465007001, Roche Diagnostics, Mannheim, Germany). The extent of alterations in MTT was quantified colorimetrically at 550 nm. The cell viability of the experimental groups was assessed with the control groups indicated as 100% viability. We performed three different experiments in triplicate for the MTT assay (Keskin et al. 2025). To examine the combined effect of Lut and Acr, different concentrations of Lut (1 and 5 μM) and Acr (1 and 2 mM) were applied to the Leydig cells, and evaluations were made according to MTT results. The potential interaction of Lut and Acr was evaluated using the SynergyFinder+ and CompuSyn software (Chou 2006; Zheng et al. 2022).

### 2.4 Lactate Dehydrogenase (LDH) Assay

TM3 Leydig cells were seeded in 96‐well plates at 1 × 10^4^ cells per well. LDH was measured using the microplate‐based Cytotoxicity Detection Kit (LDH; Cat. No: 11644793001, Roche Molecular Biochemicals, Mannheim, Germany) according to the manufacturer's instructions. The absorbance of the red formazan product was read on a spectrophotometer at 492 nm after a 30‐min incubation period. The results were validated by repeating at least three times in three separate experiments (control cells were considered 100%) (Keskin et al. 2025).

### 2.5 Biochemical Assays

For biochemical analyses, TM3 Leydig cells were seeded at 1 × 10^6^ cells per well and exposed to Lut and Acr concentrations for 24 h. After incubation, cell lysis was performed using a probe sonicator (40% amplitude) with three 10‐s pulses and 10‐s ice‐cooled intervals to prevent thermal degradation, followed by refrigerated centrifugation at 14,000 *g*; supernatants were subsequently collected and stored at −86°C for later experiments. The supernatants were used for total protein determination, malondialdehyde (MDA) amount, superoxide dismutase (SOD), catalase (CAT), glutathione peroxidase (GPx) and glutathione (GSH) measurements.

The SMART BCA protein assay kit was utilized for the colorimetric assessment and quantification of total protein using bicinchoninic acid (BCA). The purple reaction result in this assay is generated by the interaction of two BCA molecules with a copper ion. This water‐soluble complex exhibits a strong absorbance at 562 nm over a wide working range (20–2000 μg/mL) with increasing protein concentrations. The amount of membrane lipid peroxidation was measured according to the method of Heath and Packer (1968). The experiment depends on the absorbance of the pink molecule generated by the reaction between MDA, a byproduct of lipid peroxidation, and thiobarbituric acid, measured at a wavelength of 532 nm using a spectrophotometer. The SOD enzyme was measured using the method established by Marklund and Marklund (1974). This method involves the inhibition of autoxidation by pyrogallol at alkaline pH, mediated by the SOD enzyme, and is assessed using spectrophotometric measurements at 420 nm over a duration of 3 min at 30‐s intervals. The method by Sinha (1972) was employed for the determination of CAT enzyme. This technique relies on quantifying the colorimetric alteration produced by the interaction of H₂O₂ with dichromate/acetic acid at a wavelength of 570 nm. GPx activity was determined based on the absorbance of the 5′,5‐dithiobis‐(2‐nitrobenzoic acid) compound at a 412 nm wavelength according to the Hafeman et al. (1974) method. GSH determination according to the method of Ellman (1959) is based on the measurement of the absorbance of 5‐thio‐2‐nitrobenzoic acid, which is formed as a result of the reaction of GSH with 5′,5‐dithiobis‐(2‐nitrobenzoic acid), at a wavelength of 414 nm in a spectrophotometer.

### 2.6 Total ROS Assay

The technique employed by Kim and Xue (2020) was utilized to quantify the total ROS generated within the cells. In accordance with the experimental concept, DCFH‐DA is internalized by the cell, where cellular esterases cleave the acetyl group in the cytoplasm, resulting in the formation of DCFH. Subsequently, DCFH is oxidized by ROS and transforms into DCF, which exhibits fluorescent characteristics. Thus, the total ROS present in the cell is displayed in green under a fluorescent microscope. To determine total ROS, TM3 Leydig cells were plated in 24‐well culture plates at a density of 2 × 10^4^ cells. Subsequently, we administered Lut and/or Acr concentrations to the cells and incubated them for 24 h. Upon completion of the incubation time, a DCFH‐DA solution was administered to the cells at a concentration of 10 μM and incubated at 37°C for 30 min, followed by two washes with PBS. Cells were subsequently analyzed using an Olympus IX71 fluorescent microscope, and sequential images were captured with an Olympus DP72 video camera. Utilizing the ImageJ analysis software, 100 cells were evaluated from the images captured for each well, and fluorescence intensities were quantified.

### 2.7 Detection of Apoptosis by Double‐Fluorescent Staining

Live, apoptotic, and dead cells can be detected using dyes that bind to DNA and exhibit fluorescent emission. Ho342 dye allows live and apoptotic cells to be visualized in blue/purple by fluorescing under ultraviolet light, while PI dye enables dead cells to appear red/pink. For the determination of live, apoptotic, and dead cells, TM3 Leydig cells were seeded into 24‐well culture plates at a density of 2 × 10^4^ cells. After applying the determined concentrations of Lut and/or Acr to the cells for 24 h, the cells were washed with PBS. Then, 20 μL of PI dye (1 mg/mL), 20 μL of Ho342 dye (1 mg/mL), and 200 μL of an experimental mixture containing 3960 μL of PBS were added to each well and incubated at 37°C for 20 min. At the end of the incubation, the cells were washed 1–2 times with PBS. Cells exhibiting fluorescence were examined under an Olympus IX71 fluorescent attachment microscope, and serial photographs of the cells were taken with an Olympus DP72 video camera. For each group, at least 1000 cells were scanned, and live, apoptotic, and dead cells were counted, obtaining separate percentage values. The experiment was repeated three times in duplicate for each sample group (Yilmaz et al. 2017).

### 2.8 Total RNA Extraction From Cells and cDNA Synthesis

Leydig cells were seeded at 1 × 10^6^ in six‐well culture plates and exposed to Lut and/or Acr for 24 h, and then total RNA was isolated using a total RNA purification kit (Cat. No: GS5304.0100, Genaxxon, Ulm, Germany). The purity of the extracted RNA was evaluated using the Nanodrop 2000 spectrophotometer (Thermo Fisher Scientific, Wilmington). The M‐MuLV First Strand cDNA synthesis kit (Cat. No: K5147, Biomatic, ON, Canada) was utilized for the synthesis of complementary DNA (cDNA) from the extracted RNA. The purity of the synthesized cDNA was verified using the Nanodrop 2000 spectrophotometer (Yilmaz et al. 2017).

### 2.9 Quantitative Polymerase Chain Reaction (qPCR)

The qPCR methodology was employed to assess the expression levels of four genes: the antiapoptotic gene *B‐cell lymphoma 2* (*Bcl2*) and the proapoptotic genes *Bcl2‐associated X* (*Bax*), *Caspase3* (*Casp3*), and *tumor protein P53* (*Trp53*), which are implicated in apoptotic pathways. qPCR was conducted utilizing the Roche Light Cycler 480 (Roche Diagnostics, Germany). The Light Cycler 480 SYBR Green I Master kit (Cat. No: 04887352001, Roche Diagnostics, Rotkreuz, Switzerland) used in this step was used in six replicates for quantitative determination of gene expression. Thermal cycling conditions comprised an initial step at 95°C for five min, followed by 45 denaturation cycles at 95°C for 10 s, primer annealing at the melting temperature for 20 s, and extension at 72°C for 20 s. The primers used were as follows: *Bcl2* (forward: 5′‐ATGGGGTGAACTGGGGGATTG‐3′; reverse: 5′‐TTCCGAATTTGTTTGGGGCAGGTC‐3′), *Bax* (forward: 5′‐GGGTGGTTGCCCTTTTCTACT‐3′; reverse: 5′‐CCCGGAGGAAGTCCAGTGTC‐3′), *Casp3* (forward: 5′‐CTTGGTAGATCGGCCATCTGAAAC‐3′; reverse: 5′‐GGTCCCGTACAGGTGTGCTTCGAC‐3′), *Trp53* (forward 5′‐GGAGTATTTGGACGACCG‐3′; reverse: 5′‐TCAGTCTGAGTCAGGCCC‐3′), *β‐actin* (forward 5′‐CGTTGACATCCGTAAAGAC‐3′; reverse: 5′‐TGGAAGGTGGACAGTGAG‐3′). The *β‐actin* gene was designated as the reference gene for all genes, and statistical analysis was conducted utilizing the 2^−ΔΔCt^ method established by Livak and Schmittgen (2001). Primer specificity was confirmed by melt curve analysis, and all primer sets produced single distinct peaks. Additionally, primer efficiencies were validated to fall within the acceptable range of 90%–110% (Yilmaz et al. 2017).

### 2.10 Statistical Analysis

All data obtained from this study were analyzed using GraphPad Prism 10 (GraphPad Software, San Diego, CA, USA). The Shapiro–Wilk test was used to evaluate the normality of the data obtained after the experiments, and it was seen that all parameters were normally distributed. In the study, one‐way ANOVA and Tukey's multiple comparison test were used as advanced statistical analysis techniques. Results were presented as mean values accompanied by standard errors of the mean. Tests were performed with a statistical significance threshold of *p < 0.05, **p < 0.01, and ***p < 0.001.

## 3 Results

### 3.1 Lut and/or Acr Administration Inhibits Cell Viability and CI in Leydig Cells

**FIGURE 1:**
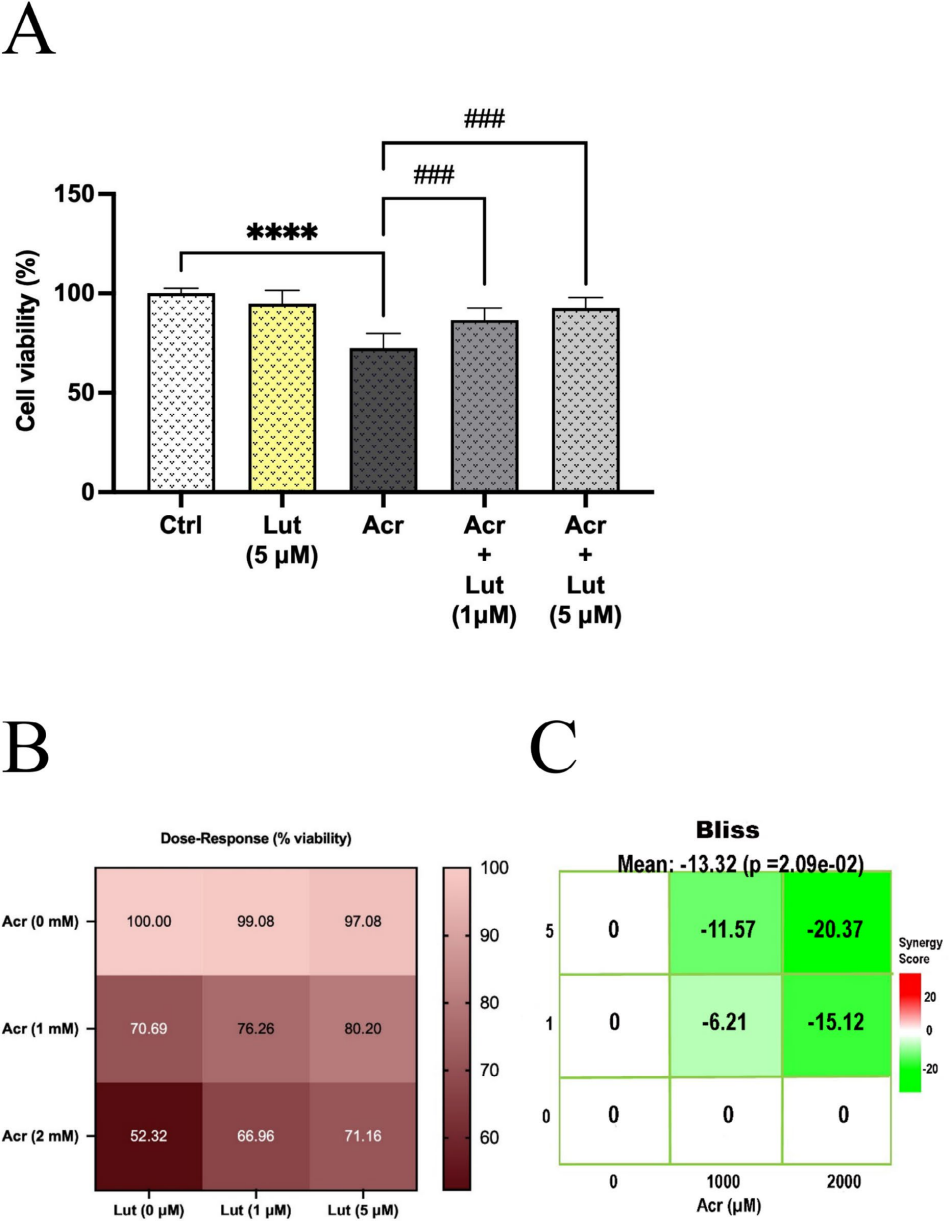
LUT improved Acr‐inhibited cell viability in Leydig cells. (A) Effects of cell viability Lut and/or Acr on Leydig cells; (B) Heat map showing the dose–response relationship of Lut and/or Acr according to % viability; (C) Lut and Acr synergy scoring (Bliss score) using minimal dose–response matrices. Data denote the mean ± SEM from three different experiments in triplicates (*n* = 9). *Indicate statistically different values in treated cells compared to the control group (****p < 0.0001) and ^#^Statistically different values in groups versus the Acr‐alone group (^###^ *p* < 0.001).

As shown in Figure 1A, the MTT results of the control group and the Acr group were compared for 24 h. It was found that in the Acr concentration, cell viability decreased to 72% (*p* < 0.0001). When the Lut + Acr groups were evaluated, it was determined that cell viability showed a significant increase to 86% in the 1 μM Lut group and to 92% in the 5 μM Lut group compared to the only Acr group (*p* < 0.001).

CompuSyn analysis represented a range of CI values for each combined concentration. The inhibition levels determined for Lut and Acr using GraphPad Prism were validated using CompuSyn and SynergyFinder+. As shown in the dose–response graph in Figure 1B, the ameliorative effects of both 1 (76.26% viability) and 5 μM (80.20% viability) Lut were detected in Leydig cells treated with 1 mM Acr (70.69% viability). Figure 1C displayed the results from the SynergyFinder+ program, which demonstrated that at concentrations of 1 (−11.57 synergy score, < 0 is antagonistic) and 5 (−6.21 synergy score, < 0 is antagonistic) μM, Lut reduced the activity of Acr when administered to Leydig cells. The combination index (CI) values calculated from a combination of Lut and Acr indicated that Lut had antagonistic effects at concentrations of 1 (CI: 1.30, moderate antagonism) and 5 μM (CI: 1.64, strong antagonism) (Table 1).

**TABLE 1:** Combination index (CI) values for combined administrations of Lut and Acr in Leydig cell line.

Acr conc. (μM)	Lut conc. (μM)	Response (% viability)	CI	Interpretation
0	0	100.00		
1000	0	70.69		
2000	0	52.32		
0	1	99.08		
1000	1	76.26	1.30	Moderate antagonistic
2000	1	66.96	1.73	Strong antagonistic
0	5	97.08		
1000	5	80.20	1.64	Strong antagonistic
2000	5	71.16	2.11	Strong antagonistic

*Note:* CI values were calculated using the Synergyfinder+ software and values defines synergism (CI < 0.9), additivity (CI = 0.9–1.1) and antagonism (CI > 1.1). Synergy/antagonism as a function of CI values (1.45–3, strong antagonism; 1.2–1.45, moderate antagonism; 0.9–1.1, additive; 0.75–0.9, slight synergistic; 0.7–0.75, moderate synergistic; 0.3–0.7, synergistic; 0.1–0.3, strong synergistic).

Abbreviations: Acr, acrylamide; Lut, luteolin.

### 3.2 Lut Suppresses Acr‐Induced Cytotoxicity in Leydig Cells

**FIGURE 2:**
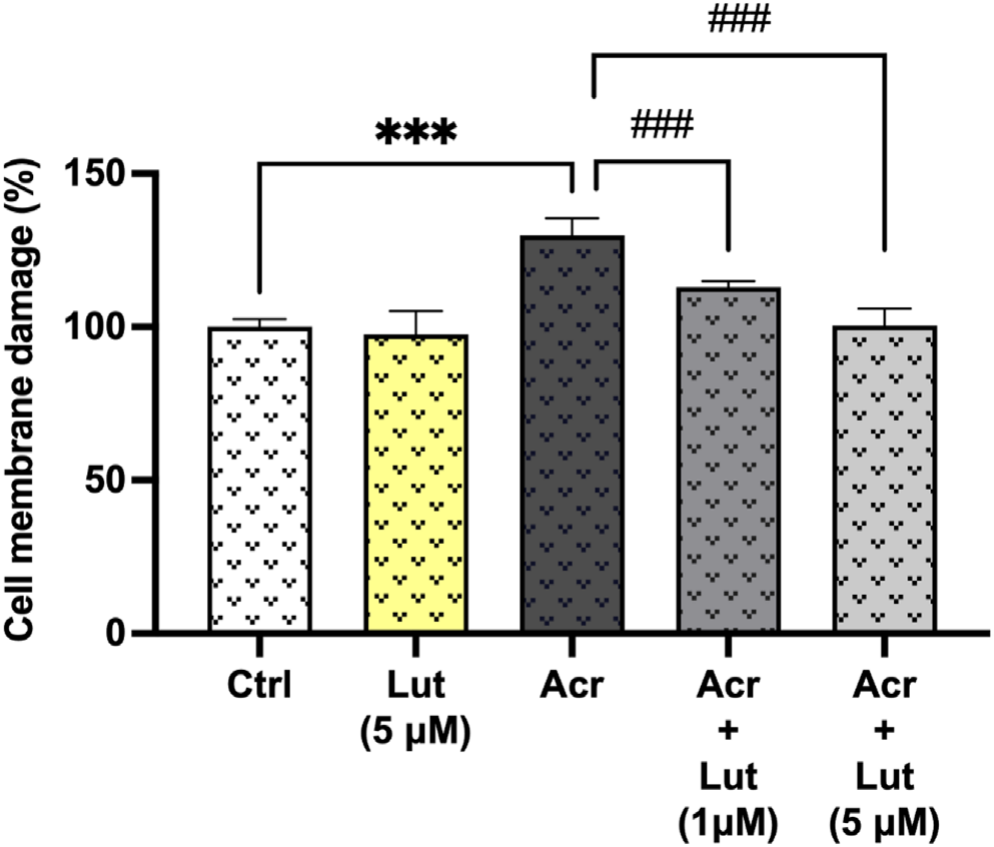
Evidence of membrane damage by LDH release in Leydig cells after treatment with Lut and/or Acr. Data denote the mean ± SEM from three different experiments in triplicates (*n* = 9). *Statistically significant differences compared to control (***p < 0.001) and ^#^Statistically significant differences compared to Acr‐only treated groups (^###^ *p* < 0.001).

The cytotoxicity test findings shown in Figure 2 indicated a substantial increase in the Acr group (130%) relative to the control group (*p* < 0.001). Comparisons between the Acr + Lut‐applied groups and the Acr‐only‐applied group revealed a reduction in the LDH rate in the Acr + Lut (1 μM, 113%) and Acr + Lut (5 μM, 100%) groups (*p* < 0.001).

### 3.3 Lut Reduces Acr‐Induced Oxidative Stress in Leydig Cells

Table 2 presented the oxidative stress findings in Leydig cells administered with Lut and/or Acr. It was found that the amount of MDA, which is the measurable end product of lipid peroxidation, increased significantly in the Acr group compared to the control group (*p* < 0.001). Furthermore, in the combined groups where two different concentrations of Lut and a single concentration of Acr were administered concurrently, increasing Lut concentrations markedly reduced MDA levels compared to the Acr group (*p* < 0.001). A significant decrease in SOD, CAT, and GPx enzyme activities was detected in the Acr group compared to the control group (*p* < 0.001). When the Acr + Lut‐applied groups were compared to the only Acr‐applied group, it was found that Lut caused a significant increase in SOD, CAT, and GPx enzyme activities (*p* < 0.01, *p* < 0.001). It was found that glutathione, a marker of oxidative stress, was inhibited by Acr (*p* < 0.05), and 5 μM Lut treatment could tolerate the inhibition caused by Acr (*p* < 0.05).

**TABLE 2:** The effects of Lut and/or Acr on oxidative stress parameters in mouse Leydig cells.

Parameters	Control	Lut (5 μM)	Acr	Acr + Lut (1 μM)	Acr + Lut (5 μM)
MDA (μmol/mg protein)	2.33 ± 0.1	2.07 ± 0.1	3.80 ± 0.1***	3.11 ± 0.1^###^	2.68 ± 0.1^###^
SOD (U/mg protein)	3.40 ± 0.2	3.27 ± 0.1	1.74 ± 0.2***	2.73 ± 0.2^##^	2.94 ± 0.2^###^
CAT (consumed nmol H_2_O_2_/min/mg protein)	0.38 ± 0.01	0.39 ± 0.01	0.30 ± 0.01***	0.34 ± 0.01^##^	0.36 ± 0.01^###^
GPx (nmol glutathione consumed/mg protein)	2.62 ± 0.1	2.68 ± 0.1	1.69 ± 0.1***	2.27 ± 0.1^##^	2.42 ± 0.1^###^
GSH (nM of GSH consumed/mg protein)	0.112 ± 0.00	0.128 ± 0.00	0.092 ± 0.00*	0.108 ± 0.0	0.113 ± 0.00^#^

*Note:* The data were given as the mean ± SEM from the three independent experiments. *p < 0.05, **p < 0.01, ***p < 0.001 compared with control. ^#^ *p* < 0.05, ^##^ *p* < 0.01, ^###^ *p* < 0.001 compared with Acr.

Abbreviations: Acr, acrylamide; CAT, catalase; GPx, glutathione peroxidase; GSH, glutathione; Lut, luteolin; MDA, malondialdehyde; SOD, superoxide dismutase.

### 3.4 Lut Scavenges ROS Generated by Acr in Leydig Cells

**FIGURE 3:**
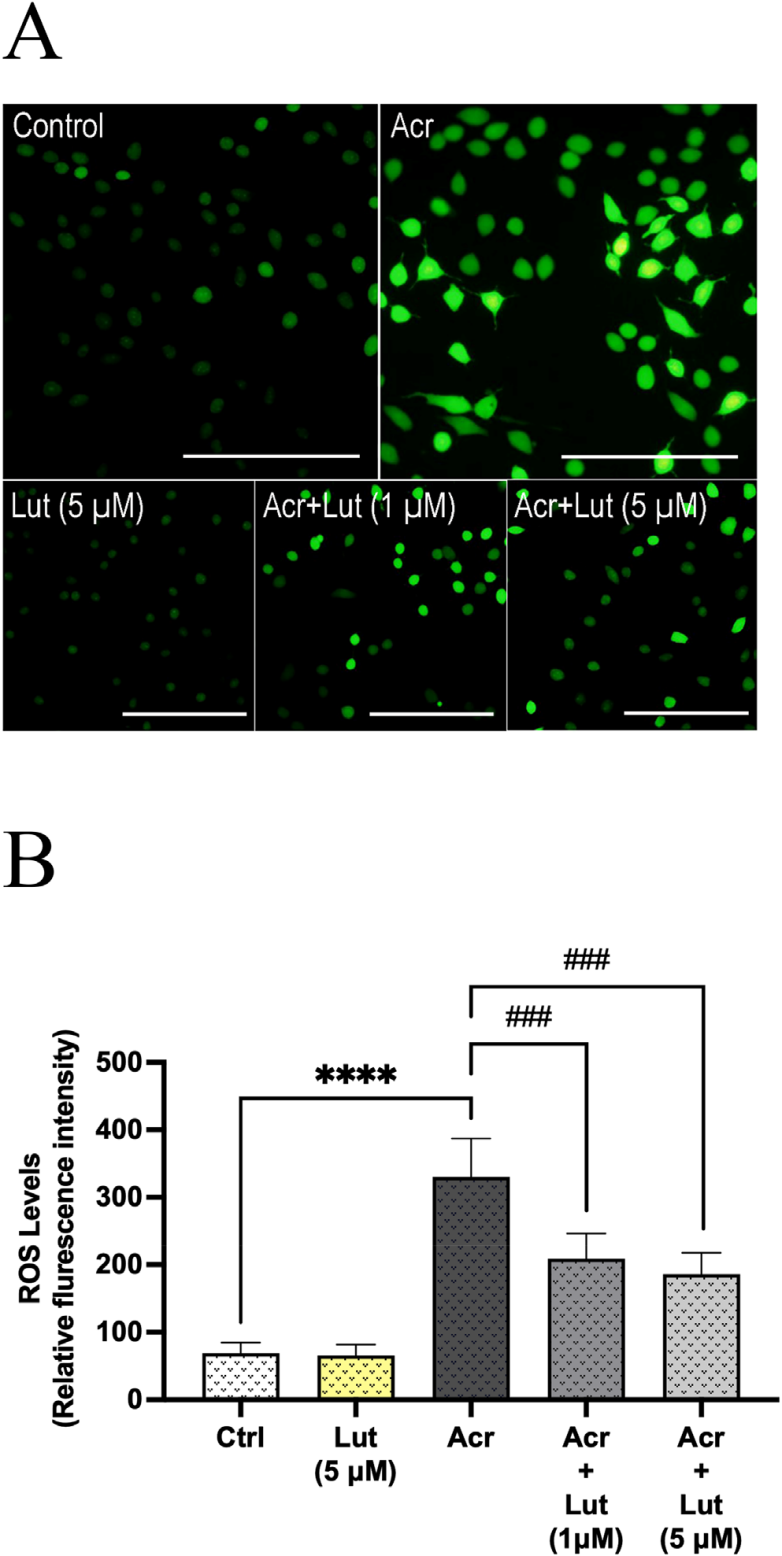
Effects of Lut and/or Acr on total ROS production in Leydig cells. (A) Representative images of Leydig cells labeled with a DCFH‐DA probe and visualized by fluorescence microscopy; (B) Total ROS levels determined by relative fluorescence intensity and expressed in arbitrary units. Experiments were repeated three times in duplicate. The values are means ± SEM (*n* = 6). *Statistically significant differences compared to control (****p < 0.0001) and ^#^Statistically significant differences compared to Acr‐only treated groups (^###^ *p* < 0.001).

In Leydig cells treated with Acr and/or Lut, the total ROS amount was presented in Figure 3 as both a representative photograph and relative fluorescence intensities, based on the emission of the DCFH‐DA dye. It was determined that the dramatic increase caused by Acr in total ROS amount was significantly reduced by Lut in both concentrations applied in addition to Acr (*p* < 0.0001).

### 3.5 Lut Modulates Acr‐Induced Apoptosis‐Related Genes in Leydig Cells

**FIGURE 4:**
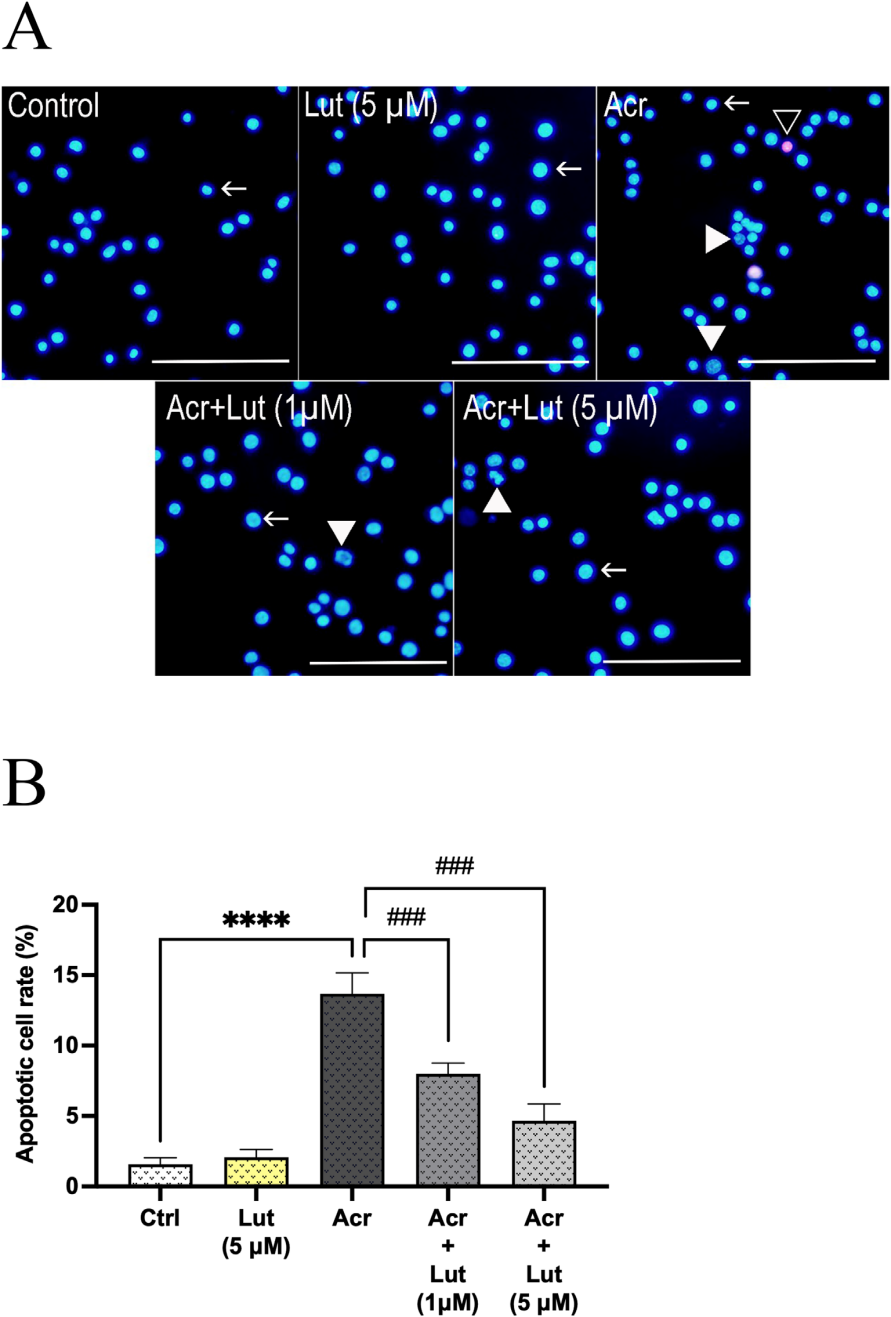
Lut prevents Acr‐induced apoptosis. (A) Double‐fluorescent labeling images with Ho342 and PI dyes are presented to show apoptosis in Leydig cells after Lut and/or Acr treatments. Arrow: live cells, write arrowhead: apoptotic cells, empty arrowhead: dead cells. Scale bar: 100 μm. (B) Effects of Lut on the apoptotic cell rate induced by Acr. Experiments were repeated three times in duplicate. The values are means ± SEM (*n* = 6). *Statistically significant differences compared to control (****p < 0.0001) and ^#^Statistically significant differences compared to Acr‐only treated groups (^###^ *p* < 0.001).

Figure 4A presented microscopy images of living, apoptotic, and dead Leydig cells following exposure to Acr and/or Lut, whereas Figure 4B illustrated the apoptotic rates. Cells were classified according to different criteria based on the data obtained with the double fluorescence staining method. Accordingly,

- Cells with bright, compact, and blue nuclei were considered live cells.
- Cells with chromatin fragmentation, nuclear condensation, and a dark blue or purple appearance were considered apoptotic cells.
- Cells without chromatin fragmentation, with a red or pink appearance, were considered dead cells.

The experimental data revealed that Acr exposure to Leydig cells significantly elevated the apoptosis rate compared to the control group (*p* < 0.0001), whereas Lut exposure combined with Acr significantly reduced the apoptosis rate relative to the Acr‐only group (*p* < 0.001).

**FIGURE 5:**
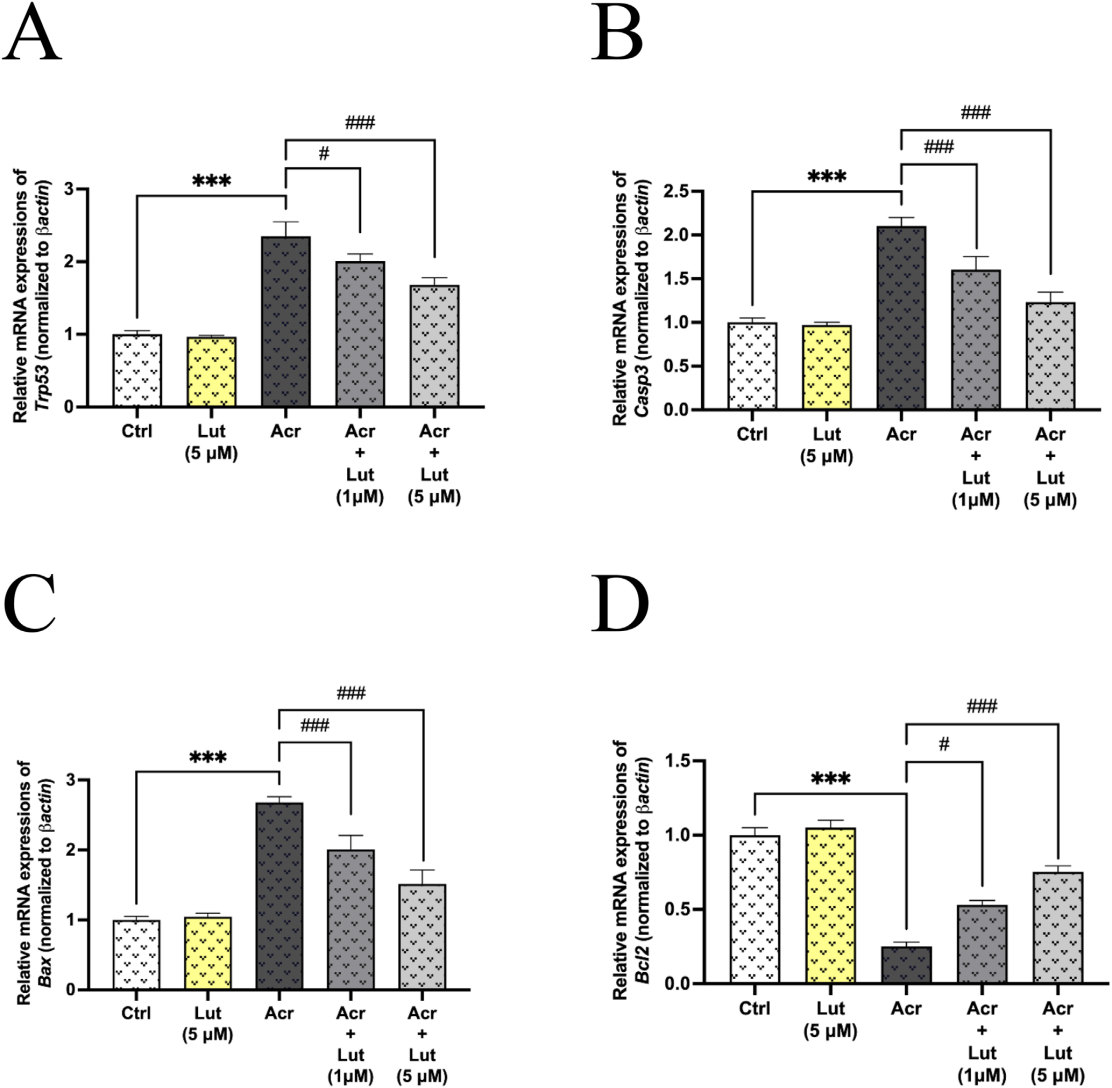
Effects of Lut and/or Acr on mRNA expression of apoptotic and antiapoptotic genes in Leydig cells. mRNA expression of (A) *Trp53*, (B) *Casp3*, (C) *Bax*, and (D) *Bcl2*. Experiments were repeated three times in duplicate. The values are means ± SEM (*n* = 6). *: Statistically significant differences compared to control (***p < 0.001) and ^#^statistically significant differences compared to Acr‐only treated groups (^#^ *p* < 0.05, ^###^ *p* < 0.001).

Figure 5 presented the effects of 1 mM Acr and 1 and 5 μM Lut combinations on the expression levels of apoptotic genes (*Trp53, Casp3, Bax*, and *Bcl2*) in Leydig cells. The Acr group exhibited elevated expression levels of *Trp53*, *Casp3*, and *Bax* genes in Leydig cells (*p* < 0.001). Conversely, increasing luteolin concentration resulted in a down‐regulation of these gene expression levels in the groups administered with both Acr and Lut, in comparison with the Acr group (Figure 5A–C, *p* < 0.05, *p* < 0.001). On the other hand, it was revealed that Acr‐inhibited *Bcl2* gene expression, while Lut suppressed this inhibition (Figure 5D, *p* < 0.05, *p* < 0.001).

## 4 Discussion

Flavonoids have protective capabilities against the effects of toxic compounds due to their different mechanisms. Flavonoids generally can prevent ROS formation, suppress oxidative stress‐induced genome instability, and mitigate DNA damage. Lut, an important member of flavonoids, provides protection against chemical and natural toxic substances with its antioxidant, anti‐inflammatory, and antiapoptotic properties (Mahdiani et al. 2022). Recent research indicates that the antioxidant properties of Lut could reduce the effects of numerous harmful substances (Alekhya Sita et al. 2019; Dar et al. 2021; Rajput et al. 2021). While numerous in vivo and in vitro studies demonstrate the protective effects of various antioxidants against the detrimental effect of Acr on the male reproductive system (Sengul et al. 2023; Sun et al. 2018; Üremiş et al. 2024; Yildizbayrak and Erkan 2019), there is a lack of research examining the effects of Lut on Acr‐induced toxicity (Keskin et al. 2025; Nabil et al. 2024). In this study, the protective effects of Lut on Acr‐induced toxicity in Leydig cells were revealed by examining cytotoxicity, oxidative damage, and apoptosis parameters.

Numerous studies examine the ameliorative effects of various antioxidant molecules on Acr‐induced cytotoxicity in different cell lines (Albalawi et al. 2017; Keskin et al. 2025; Sun et al. 2018; Yildizbayrak and Erkan 2019). In studies conducted with Leydig and Sertoli cells, which are the basic cells of the male reproductive system, it has been shown that Acr inhibits cell viability and increases LDH levels at various concentrations (Sun et al. 2018; Yildizbayrak and Erkan 2019; Yilmaz et al. 2017; Zhang et al. 2023). Moreover, research has demonstrated that antioxidant substances, including allicin, carnosic acid, and cyanidin‐3‐glucoside, may alleviate Acr‐induced cytotoxicity (Albalawi et al. 2017; Hong et al. 2019; Song et al. 2013). Similarly, our findings regarding cell viability and LDH enzyme activity in this study showed that Lut can inhibit Acr‐induced cytotoxicity. Furthermore, the investigation of the potential interaction between Lut and Acr revealed that Lut demonstrated an antagonistic effect on Acr, which was notably pronounced at a concentration of 5 μM. The results suggest that Lut may serve as a possible therapeutic agent in Acr cytotoxicity. Acr can induce oxidative damage by causing excessive production of ROS in the normal metabolic processes of cells (Hong et al. 2021; Salimi et al. 2022). Due to increased ROS levels, biological molecules such as lipids, proteins, and DNA are damaged, resulting in cellular impairment (Albalawi et al. 2017). Lipids, which are important biological molecules, constitute the basic components of cell membranes, and ROS cause the formation of lipid peroxides. Increased MDA levels arise from lipid peroxidation, disrupting cell membrane integrity (Całyniuk et al. 2016). The biological defense systems play an active role in the reorganization of cellular homeostasis. Antioxidant enzymes, including SOD, CAT, and GPx, function to protect against the detrimental effects of ROS. Various chemicals and food contaminants reduce the levels of antioxidant enzymes in organisms, causing the oxidative balance to deteriorate. Research indicates that Acr, utilized as an industrial chemical and generated by heating in food, influences the levels of antioxidant enzymes (Albalawi et al. 2017; Orta‐Yilmaz 2019). Numerous in vitro studies have examined the impact of Acr on ROS‐induced lipid peroxidation and the protective action of various antioxidant agents against these effects (Albalawi et al. 2017; Jiang et al. 2018; Yildizbayrak and Erkan 2019). This in vitro study with Leydig cells demonstrated that the dramatic increase in ROS and lipid peroxidation induced by Acr prevented cellular damage by showing strong healing properties at both concentrations of Lut (1 and 5 μM). In some studies, showing that Acr inhibits GSH, it has been found that carnosic acid in human retinal pigment epithelium cells, cyanidin‐3‐glucoside in human breast cells, and lipoic acid in microglial cells improve GSH levels (Albalawi et al. 2017; Song et al. 2013; Song et al. 2017). Consistent with these in vitro investigations, in our study conducted with Leydig cells, it was determined that Lut was effective in improving GSH levels. Many in vitro experiments have examined the effect of Acr on antioxidant enzyme activities (SOD, CAT, GPx) and the therapeutic potential of molecules such as allicin, curcumin, Ganoderma, and carnosic acid in neutralizing these effects (Albalawi et al. 2017; Hong et al. 2019; Jiang et al. 2018; Yildizbayrak and Erkan 2019). The data obtained from our study are similar to the studies conducted with these different antioxidants in the literature, and it has been revealed that Lut may also be an effective molecule in reversing the effects of Acr on SOD, CAT, and GPx enzyme activities.

Apoptosis is regulated by multiple pathways and is considered a consequence of oxidative stress resulting from elevated ROS levels (Asadi et al. 2021). Acr significantly elevated ROS levels in many cell lines, thereby triggering apoptotic signals and resulting in cell death (Albalawi et al. 2017; Jiang et al. 2018; Keskin et al. 2025; Sun et al. 2018; Yildizbayrak and Erkan 2019; Yilmaz et al. 2017). The effect of Acr on the expression levels of specific genes associated with apoptosis (*Trp53*, *Casp3*, *Bax*, and *Bcl2*) has been established by multiple in vitro and in vivo investigations (Abd‐Elsalam et al. 2021; Ajibare et al. 2024; Sengul et al. 2023; Yilmaz et al. 2017). Research showed that the altered expressions of *Bax*, *Bcl2*, *Casp3*, and *Casp9*, in rat small intestine cells treated with Acr, were enhanced by the administration of Ganoderma (Jiang et al. 2018). A study by Albalawi et al. (2017) established that the *Casp3* and *Casp9* genes, which were elevated by Acr exposure, may be downregulated by carnosic acid. The administration of naringin was shown to protect cells against apoptosis by inhibiting the production of the Acr‐induced *Trp53* gene (Sengul et al. 2023). A study indicated that a combination of Acr and zinc was found to increase the decreased *Bcl2* levels and significantly decrease the increased *Bax* levels, thus suppressing apoptosis (Ajibare et al. 2024). This study by using Leydig cells revealed that Acr elevated the mRNA expression levels of the *Bax*, *Casp3*, and *Trp53* genes, while reducing the expression level of the *Bcl2* gene. For the first time at the gene level, it has been demonstrated that Lut exerts protective effects against apoptosis by altering the expression levels of apoptotic and antiapoptotic genes, similar to the findings associated with carnosic acid, naringin, and zinc in diminishing Acr‐induced apoptosis as reported in the literature.

This study meticulously examined the protective effects of Lut against Acr‐induced cellular damage in vitro. However, the study has some limitations. Although this study demonstrated the protective effects of Lut against Acr‐induced toxicity in mouse Leydig cells, the findings are based on an in vitro model and should be interpreted with caution in terms of translational validity. In vitro systems provide controlled environments for the investigation of cellular mechanisms; however, they do not fully reflect the complexity of living organisms, such as metabolism, systemic interactions, and bioavailability. Therefore, advanced in vivo studies are required to confirm the efficacy and safety of Lut against Acr‐related reproductive toxicity under physiological conditions. Another limitation is that signaling pathways related to oxidative stress and apoptosis (e.g., Nrf2/HO‐1 and MAPK/NF‐κB) were not included in our study. The lack of analyses at both the gene and protein levels to directly confirm the involvement of signaling cascades left mechanistic inferences limited. While the enzyme and gene expression data examined in our study provide indirect support, additional studies are needed to more clearly define the molecular mechanisms behind the protective effects observed with Lut treatment. Incorporating such methods into future studies will improve mechanistic insight and increase the potential relevance of the findings to biological systems.

## 5 Conclusion

Our findings showed that Lut has the potential to alleviate Acr‐induced oxidative stress damage and apoptosis in Leydig cells. This protective effect of Lut may be achieved through its ability to act antagonistically against Acr, maintain membrane permeability, increase antioxidant enzyme levels, and regulate the expression levels of proapoptotic and antiapoptotic genes. Interestingly, Lut prevents apoptosis by increasing cell viability, the expression of the antiapoptotic *Bcl2* gene, and the activity of intracellular antioxidants, suppressing total ROS formation and the expression of the proapoptotic *Trp53, Casp3*, and *Bax* genes. Therefore, it seems reasonable that Lut may function as a powerful protective molecule against Acr‐induced cellular damage in our cells, which serve as a model for the male reproductive system.

## Author Contributions

**Ertan Calmaz:** investigation (supporting), methodology (supporting). **Banu Orta‐Yilmaz:** conceptualization (equal), formal analysis (equal), investigation (equal), methodology (equal), validation (equal), visualization (equal), writing – original draft (equal), writing – review and editing (equal). **Yasemin Aydin:** conceptualization (equal), formal analysis (equal), funding acquisition (equal), investigation (equal), methodology (equal), project administration (equal), supervision (equal), validation (equal), visualization (equal), writing – original draft (equal), writing – review and editing (equal).

## Ethics Statement

The authors have nothing to report.

## Conflicts of Interest

The authors declare no conflicts of interest.

## Data Availability

The data that support the findings of this study are available from the corresponding author upon reasonable request.
